# Extraskeletal osteosarcoma in the rectus abdominis muscle: a case report

**DOI:** 10.1093/jscr/rjag294

**Published:** 2026-04-23

**Authors:** Kazunori Nakayama, Hideki Ueda, Kento Fukaya, Katsumi Shimomura, Eiichi Konishi, Toru Osawa

**Affiliations:** Department of Orthopedic Surgery, Japanese Red Cross Society Kyoto Daiichi Hospital, 15-749 Honmachi Higashiyama-ku, Kyoto 605-0981, Japan; Department of Orthopedic Surgery, Japanese Red Cross Society Kyoto Daiichi Hospital, 15-749 Honmachi Higashiyama-ku, Kyoto 605-0981, Japan; Department of Orthopedic Surgery, Saiseikai Shiga Hospital, 2-4-1 Ohashi Ritto, Shiga 520-3046, Japan; Department of Rehabilitation Medicine, Kyoto Prefectural University of Medicine, 465 Kajii-cho, Kamigyo-ku, Kyoto 602-8566, Japan; Department of Surgical Pathology, Kyoto Prefectural University of Medicine, 465 Kajii-cho, Kamigyo-ku, Kyoto 602-8566, Japan; Department of Orthopedic Surgery, Japanese Red Cross Society Kyoto Daiichi Hospital, 15-749 Honmachi Higashiyama-ku, Kyoto 605-0981, Japan

**Keywords:** extraskeletal osteosarcoma, rectus abdominis muscle, denosumab, giant cell tumor

## Abstract

Extraskeletal osteosarcoma is a rare malignant tumor with an osteoid or bone matrix that develops in soft tissues that are not in contact with bone or periosteum. A 47-year-old woman presented with a subcutaneous mass in her right abdomen. A needle biopsy revealed a giant cell tumor of the soft tissue. Denosumab was administered, but the tumor enlarged. Wide tumor resection was performed. Histopathological analysis confirmed the diagnosis of extraskeletal osteosarcoma. The patient remains alive and disease-free one year after the surgery. In this case, based on the results of the needle biopsy, chemotherapy was not administered, and surgery was prioritized. Because the tumor has a poor prognosis, careful follow-up is recommended and will be continued.

## Introduction

Extraskeletal osteosarcoma is a malignant tumor with an osteoid or bone matrix that develops in soft tissues that are not in contact with bone or periosteum [1]. There have been very few reports of cases occurring in the abdomen. We report an extremely rare case of extraskeletal osteosarcoma occurring within the right rectus abdominis muscle.

## Case presentation

**Figure 1:**
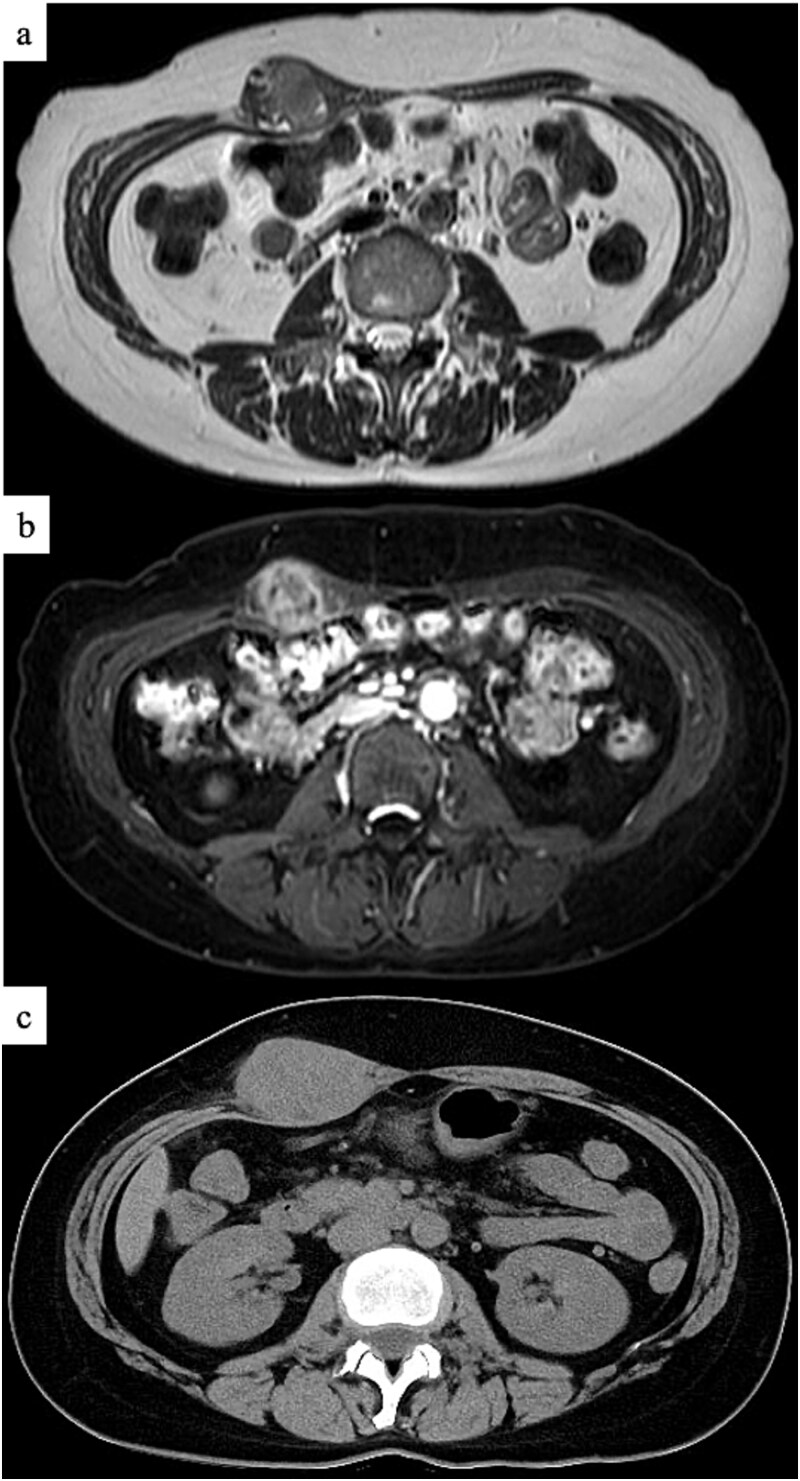
MR images showing a mass in the right abdominal wall. (a) T2WI, (b) Gadolinium (Gd) enhanced. (c) CT image showing a homogeneous isodense lesion without calcification on the right abdominal wall.

**Figure 2:**
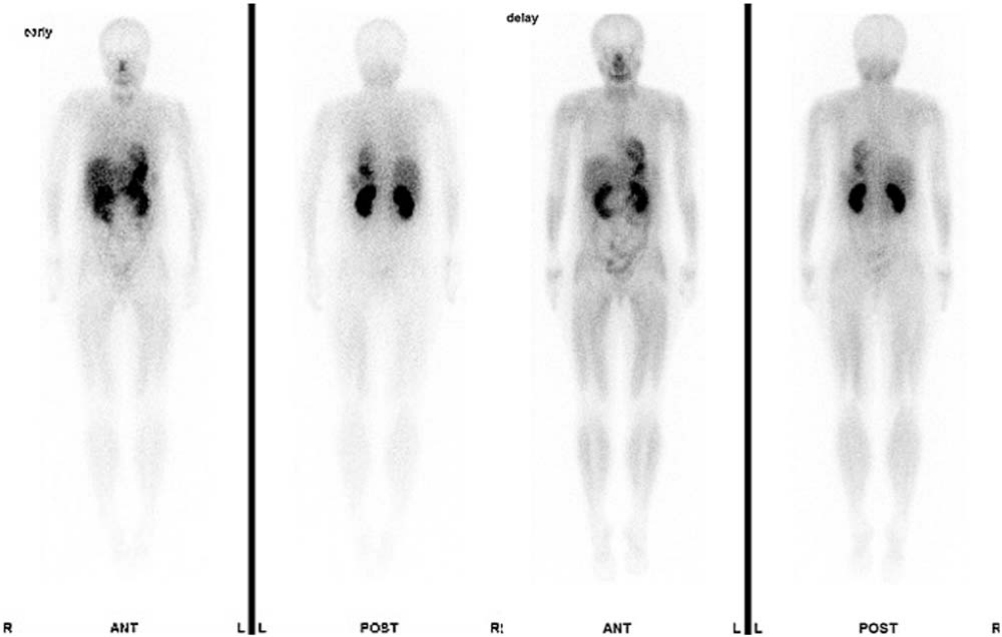
Thallium-201 scintigram showing uptake in the early phase and no wash-out in the late phase.

**Figure 3:**
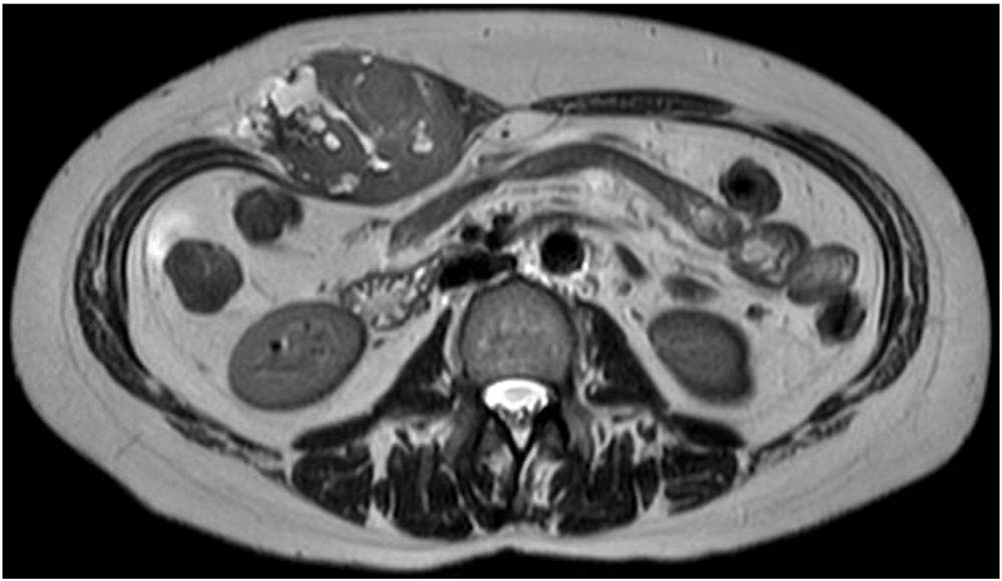
T2WI MR image showing an expanding mass after denosumab administration.

**Figure 4:**
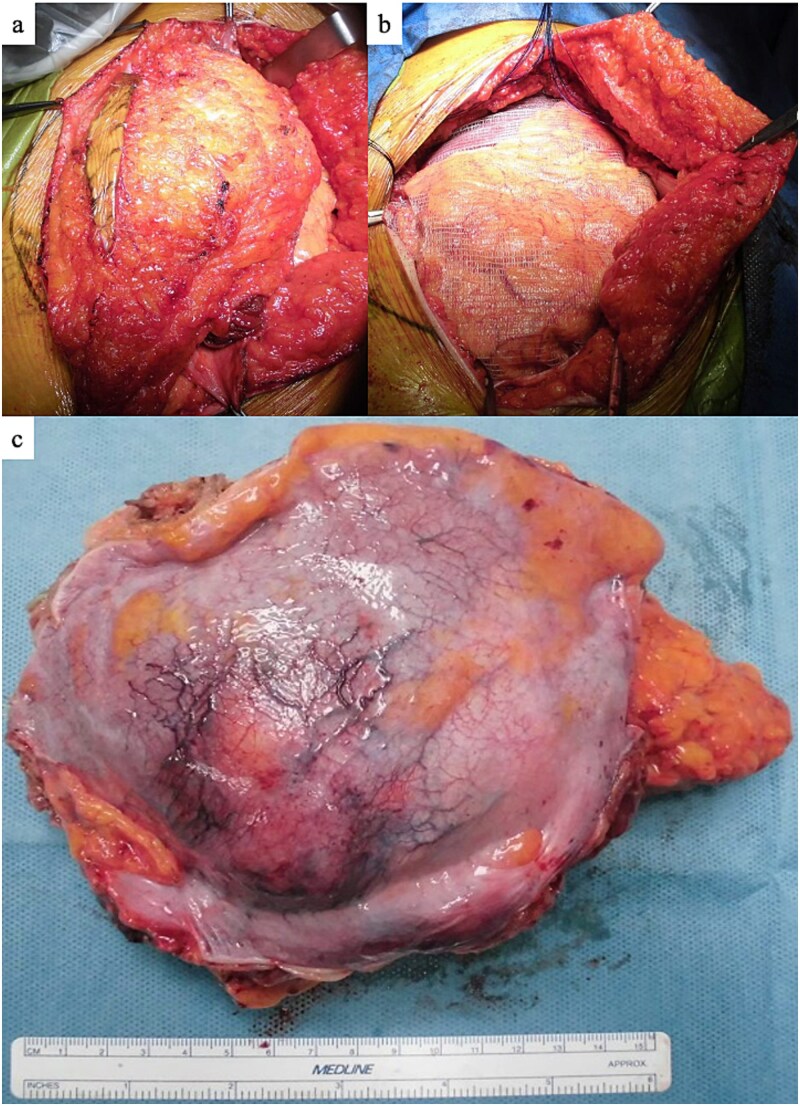
Operation findings. (a) Abdominal wall resection with a margin of approximately 3 cm from the tumor. (b) The abdominal wall defect reconstructed with ORIHIME^®^ mesh. (c) Tumor on the peritoneal side. No adhesions were observed in the abdominal cavity.

**Figure 5:**
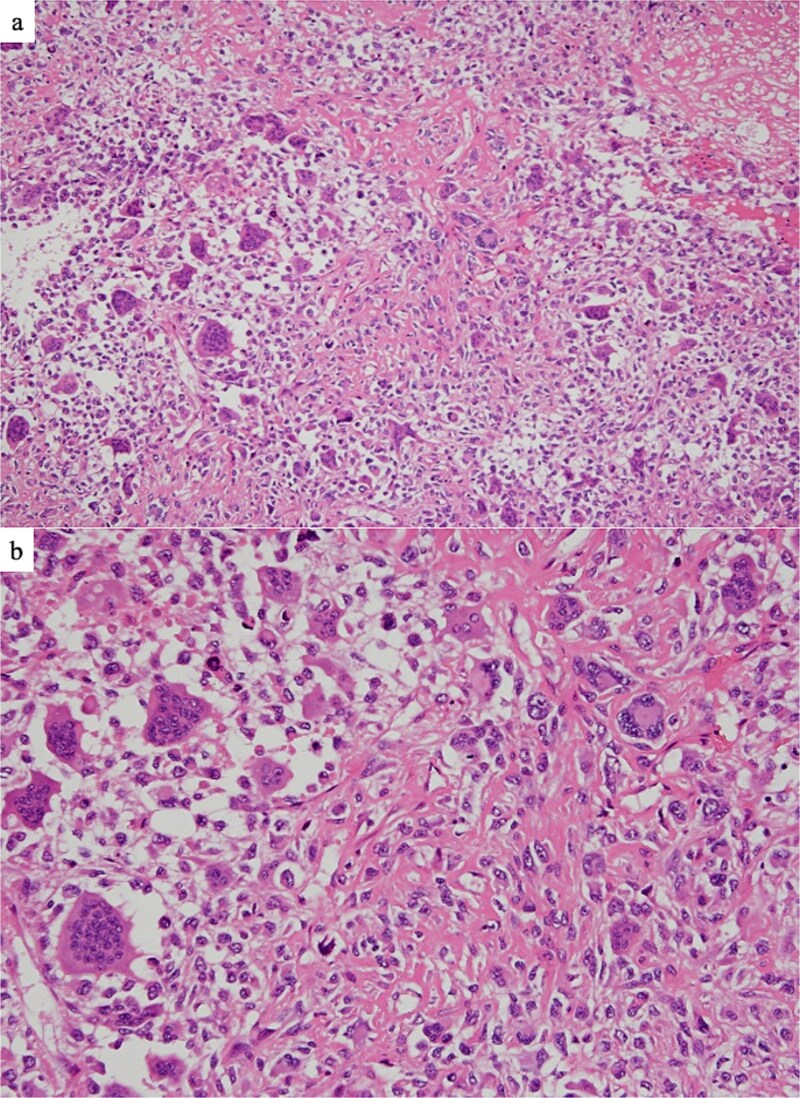
Histopathological findings. (a) The stroma is collagenous and fibrous, with edematous and osteoid parts showing vitrification (Hematoxylin and eosin staining, original magnification 40×). (b) Strong nuclear atypia of tumor cells with large and irregular nuclei and numerous mitoses. Osteoclast-like or foreign body-type giant cells were observed (hematoxylin and eosin staining, original magnification 200×).

A 47-year-old woman presented to the dermatology department of our hospital with a subcutaneous mass in her right abdomen. Ultrasonography revealed a mass in the right rectus abdominis muscle, and the patient was referred to our department. On local examination, a 3-cm-sized, elastic, hard, and mobile mass with tenderness was palpated. Contrast-enhanced magnetic resonance (MR) images showed a lesion measuring 30 × 32 × 35 mm in diameter with isointensity on T1-weighted images, high intensity on T2-weighted images (Fig. 1a), and an enhanced contrast effect (Fig. 1b). Because the patient had a history of endometriosis, they were suspected to have ectopic endometriosis and were referred to the gynecology department of our hospital where a needle biopsy was performed. Histopathological examination revealed a giant cell tumor of the soft tissue. Computed tomography (CT) showed a homogeneous isodense lesion without calcification (Fig. 1c). Thallium-201 scintigraphy showed uptake in the early phase, but no wash-out in the late phase (Fig. 2). We administered denosumab treatment. However, the tumor continued to expand, and MR images showed that the tumor was >6 cm in diameter (Fig. 3). Wide tumor resection was performed. The abdominal wall was resected with a margin of approximately 3 cm from the tumor (Fig. 4a–c). No adhesions were observed in the abdominal cavity. The abdominal wall defect was reconstructed using an ORIHIME^®^ mesh. Histopathologically, the tumor was classified as a pleomorphic sarcoma consisting of mononuclear and atypical spindle cells admixed with osteoclast-type giant cells. The tumor stroma was collagenous with focal and osteoid formation. The nuclear atypia of tumor cells was severe with large and irregular nuclei and numerous mitoses (Fig. 5a and b). Therefore, the tumor was diagnosed as extraskeletal osteosarcoma. Although the tumor had invaded the vicinity of the inner abdominal wall, it was determined that an R0 resection was performed. Chemotherapy and radiotherapy were not performed. Currently, the patient remains alive and disease-free one year after the surgery.

## Discussion

Extraskeletal osteosarcomas are extremely rare tumors that account for 1% of all malignant soft tissue tumors and 4% of osteosarcomas [1]. The most common sites of disease are deep, proximal to the lower extremities (46.6%), upper extremities (20.5%), or retroperitoneum (17%) [2]. Longhi *et al*. [3] reported that abdominal cases occurred in only 8 of 216 extraskeletal osteosarcoma cases, indicating the rarity of abdominal occurrences. Extraskeletal osteosarcoma occurs in slightly older patients than conventional osteosarcoma, and most cases occur in patients between the ages of 50 and 60 years old [1]. The male-to-female ratio is 1.9:1 [1]. A history of trauma or radiation to the site of the eventual tumor was found in 16 of 88 cases in a prior study (18.2%) [2]. The prognosis for patients with extraskeletal osteosarcoma is poor, with a 5-year survival rate of 25% or less [1]. Histopathologically, osteosarcoma is a highly active sarcoma that is highly cellular and comprises pleomorphic, mitotic, and epithelioid spindle cells [1]. It can be confirmed by the presence of neoplastic osteoid or bone. Osteoclast-like giant cells are also observed. In addition, it has been reported that, immunohistologically, extraskeletal osteosarcoma is similar to conventional osteosarcoma and is positive for vimentin; however, osteosarcomas are less frequently positive for other proteins such as SMA (68%), desmin (25%), and S-100 protein (20%) [1]. There are no defining imaging features, and MR imaging shows isointensity on T1-weighted images, high intensity on T2-weighted images, and heterogeneous enhancement on gadolinium-enhanced images [4]. Calcification is present in less than one-third of conventional osteosarcomas [4]. Tc-99mMDP radionuclide scans may show increased uptake in both the primary lesion and the metastatic foci, which is consistent with osteoblastic activity within the tumor [5]. Similarly, 18F-fludeoxyglucose (FDG) positron emission tomography images often show intense FDG-avid foci in both the primary and metastatic lesions [6]. Regarding the treatment of extraskeletal osteosarcoma, chemotherapy for conventional osteosarcoma is reported to be more effective than chemotherapy for soft tissue sarcoma [3]. However, as the response is not as good, surgical resection is the most common treatment method for extraskeletal osteosarcoma [7]. Furthermore, Wang *et al*. did not identify any survival benefit in patients with extraskeletal osteosarcoma who received different chemotherapy regimens or between patients who received chemotherapy and those who did not receive chemotherapy [8]. Longhi *et al*. [3] reported that radiotherapy was an adjuvant treatment and was frequently used in combination with R1 resection; it was found to be effective in R0 resection cases with tumor diameters between 5 and 10 cm. However, adjuvant radiotherapy was ineffective in R1 resection when surgery with inadequate margins was performed. In contrast, Wang *et al*. reported that radiation therapy improved the overall survival for patients who were unable to achieve negative margins (R1 and R2) [8].

In this case, based on the results of the needle biopsy, chemotherapy was not administered, and surgery was prioritized. Fortunately, no recurrence or metastasis has been observed. However, because the tumor has a poor prognosis, careful follow-up is recommended and will be continued.

## Data Availability

The datasets used and/or analyzed in the current study are available from the corresponding author upon reasonable request.
